# Hereditary Breast Cancer in the Han Chinese Population

**DOI:** 10.2188/jea.JE20120043

**Published:** 2013-03-05

**Authors:** Wenming Cao, Xiaojia Wang, Ji-Cheng Li

**Affiliations:** 1 Institute of Cell Biology, Zhejiang University, Hangzhou, China; 2 Division of Oncology, Zhejiang Cancer Hospital, Hangzhou, China

**Keywords:** hereditary breast cancer, breast cancer susceptibility, high-penetrance genes, moderate-penetrance genes, Chinese

## Abstract

Breast cancer is the most common malignancy among women and has a strong genetic background. So far, 13 breast cancer susceptibility genes of high or moderate penetrance have been identified. This review summarizes findings on these genes in Han Chinese. *BRCA1* and *BRCA2* are the 2 most important susceptibility genes. They have a relatively low mutation rate, and the most frequent sites of mutation are in exon 11. Frameshift mutations are the main type of mutation. Founder mutations may also exist, and *BRCA*-associated breast cancer has specific clinicopathologic characteristics. *TP53* and *PALB2* are relatively rare susceptibility genes. The relationship between the other 9 genes and breast cancer has not been fully elucidated. At present, the mutation spectrum for these susceptibility genes is not well understood in the Chinese population, and there are few reports on prognosis and clinical intervention in high-risk populations. Therefore, the true value of genetic counseling for breast cancer has yet to be realized. This article reviews studies of hereditary breast cancer in the Han Chinese population, highlights potential inadequacies, and provides a foundation for genetic counseling for breast cancer in China.

## 1. INTRODUCTION

Breast cancer has an incidence rate of 16.39 per 100 000 Chinese women and seriously affects the lives and health of this population. Among women in economically developed Chinese provinces and cities, breast cancer has the highest incidence of all cancers and is the fourth most common cause of cancer death. Breast cancer also has a strong genetic background. Hereditary breast cancer tends to display familial aggregation and is associated with early age at onset and a high incidence of bilateral occurrence. Since the discovery of the breast cancer susceptibility genes *BRCA1* and *BRCA2* in 1994, a total of 18 breast cancer-associated susceptibility genes have been identified. These genes include breast cancer susceptibility genes with high penetrance (*CDH1*, *NBS1*, *NF1*, *PTEN*, *TP53*, and *STK11*), moderate penetrance (*ATM*, *BRIP1*, *CHEK2*, *PALB2*, and *RAD50*), and low penetrance (*FGFR2*, *LSP1*, *MAP3K1*, *TGFB1*, and *TOX3*).^1^ The United States and most countries in Europe have professional genetic services programs that provide genetic testing and counseling regarding the risk of developing breast cancer. Research on hereditary breast cancer in the Chinese population started late, and relevant studies of mainland Chinese have only begun to be reported in the last decade or so. However, most of these studies involved single centers and small numbers of cases, which are important shortcomings. China has 56 ethnic groups, but the Han ethnic group makes up more than 90% of the country’s population. Therefore, most studies of hereditary breast cancer have focused on this population, to the exclusion of other ethnic groups. Moreover, although several single nucleotide polymorphisms (SNPs) associated with breast cancer risk have been identified as low-penetrance susceptibility polymorphisms within genes, the frequencies of these polymorphisms substantially differ between races, which leads to inconsistent results. For example, the *TGFRB1* rs1982073 C-allele increases breast cancer risk in many races^2^ but is not associated with breast cancer risk in the Chinese population.^3^ Eight SNPs (rs2046210, rs1219648, rs3817198, rs8051542, rs3803662, rs889312, rs10941679, and rs13281615) are associated with breast cancer risk among Chinese women, and a risk assessment model that includes these genetic markers and clinical predictors might be useful in classifying Asian women into relevant risk groups.^4^ However, the moderate discriminatory accuracy provided by such a full risk assessment model was inadequate for cancer diagnosis and screening. In addition, absolute risk estimates generated by the model would be applicable only to populations with rates comparable to those seen in Shanghai. Therefore, further research is needed to evaluate low-penetrance susceptibility genes and their association with breast cancer risk in the Chinese population. Currently, it is too early for low-penetrance susceptibility genes to be described as the target of genetic counseling in Chinese population. Therefore, we do not discuss such genes in this article. Instead, we have chosen to review studies of moderate- and high-penetrance breast cancer susceptibility genes in the Han Chinese population.

## 2. *BRCA1* AND *BRCA2*

*BRCA1* and *BRCA2* are located on chromosomes 17q21 and 13q12.3, respectively. *BRCA1* consists of 24 exons, of which exons 1 and 4 are non-coding. *BRCA2* consists of 27 exons, of which exon 1 is non-coding. The BRCA1 and BRCA2 proteins have an important role in repairing DNA double-stranded breaks. Germline mutations in these 2 genes contribute to the pathogenesis of 20% to 40% of familial breast cancers in whites, thus accounting for 5% of all breast cancers.^5^ The risk of breast cancer in carriers of the *BRCA1* and *BRCA2* mutations is 51% to 75% and 33% to 54%, respectively, before age 70 years. In particular, there is a 100-fold higher risk of breast cancer in male carriers of the *BRCA2* mutation.^6^ These 2 genes have been frequently reported in the Chinese population. *BRCA1* and *BRCA2* germline point mutations in the Han Chinese population are shown in eTables 1 and 2, respectively.

### 2.1 Point mutation and founder mutation

Point mutations are the most common type of mutation and have been frequently reported in the Chinese population. Mutations within exon 11 of *BRCA1* and *BRCA2* are the most common and accounted for 56.3% (58/103) and 53.8% (49/91) of all mutations, respectively (Tables 1 and 2). The most common detection methods include polymerase chain reaction (PCR)-based screening methods such as single-strand conformation polymorphism (SSCP) and denaturing high-performance liquid chromatography (DHPLC). After detection of abnormalities and subsequent sequencing, these methods can significantly reduce testing costs, but a considerable number of disease-associated mutations may be missed by these indirect detection methods.^24^ In particular, the sensitivity of SSCP is a concern, as it has a direct-sequencing mutation detection rate of 76%. The mutation types are frameshift mutation, nonsense mutation, splice-site mutation, and missense mutation. Frameshift mutation is the most common, accounting for 63.1% (65/103) and 74.7% (68/91) of all mutations in *BRCA1* and *BRCA2*, respectively (Tables 1 and 2). Frameshift mutations, nonsense mutations, and the effects of splicing can cause abnormal protein function and are the most easily identified pathogenic mutations. However, further verification is required for some other site changes. For example Kwong et al^25^ reported that the 7806-9T>G mutation at *BRCA2* intron 16 can cause 3 types of aberrant splicing variants: 7806_7874del, 7806_7976del, and 7806-8_7806-1ins. All identified cut protein was truncated; thus, the 7806-9T>G mutation is pathogenic. Some missense mutations are also pathogenic, eg, 5482G>T in *BRCA1* can cause Gly1788Val,^17^ as has been reported in the Breast Cancer Information Core (BIC).

**Table 1.** Disease-associated *BRCA1* germline mutations in Chinese with breast/ovarian cancer

Author (reference)	Year published	Techniques	Types of cases (No. of cases)	Mutation positions (No. of cases)	Mutation types (No. of cases)
Li et al^7^	1999	PCR-SSCP-SEQ	HBC (18)	Intron7 (2)	SS (2)
Sng et al^8^	2000	PCR-SSCP-SEQ	HBC (16)	Exon11 (1), Exon13 (1)	FS (1), NS (1)
			Early-BC (60)	Exon11 (4)	FS (4)
Zhi et al^9^	2002	PCR-SSCP-SEQ	HBC (16)	Exon11 (1)	NS (1)
			Early-BC (20)	Exon16 (1)	FS (1)
Deng et al^10^	2003	PCR-DHPLC-SEQ	HBC (9)	Exon11 (1)	FS (1)
Suter et al^11^	2004	PCR-SSCP-SEQ	HBC + SBC (645)	Intron2 (2), Exon11 (3), Exon24 (2)	FS (5), SS (2)
Zhou et al^12^	2004	PCR-SEQ	HBC + Early-BC (14)	Exon11 (1)	NS (1)
Li et al^13^	2006	PCR-DHPLC-SEQ	HBOC + Bi-BOC (25)	Exon9 (1), Exon11 (5), Exon12 (1), Exon24 (3)	FS (7), NS (3)
Huang et al^14^	2008	PCR-DHPLC-SEQ	HBC (19)	Exon6 (1)	NS (1)
Thirthagiri et al^15^	2008	PCR-SEQ	HBOC (78) + Early-BC (40)	Exon2 (1), Exon8 (1), Exon11 (2), Exon13 (1), Intron3 (2), Intron14 (1)	FS (4), NS (1), SS (3)
Li et al^16^	2008	PCR-DHPLC-SEQ	HBC (261)	Intron3 (1), Exon10 (1), Exon11 (9), Intron16 (1), Intron21 (1), Exon24 (3)	FS (10), NS (3), S (3)
			Early-BC (228)	Exon8 (1), Exon11 (4), Exon24 (2)	FS (4), NS (3)
			SBC (426)	Exon11 (1), Exon24 (2)	FS (3)
Chen et al^17^	2009	PCR-DHPLC-SEQ	HBC (68)	Exon11 (1), Intron21 (1), Exon22 (1), Exon24 (1)	FS (2), MS (1), SS (1)
			Early-BC (71)	Exon11 (2)	FS (2)
Zhou et al^18^	2009	PCR-SEQ	Early-BC (41)	Exon5 (1)	MS (1)
Kwong et al^19^	2009	PCR-SEQ	High-risk BC + OC (119)	Intron5 (1), Exon8 (1), Exon11 (4), Exon14 (1)	FS (3), NS (3), SS (1)
Xue et al^20^	2010	PCR-SSCP-SEQ	HBC (54)	Exon5 (1), Exon11 (2)	FS (2), NS (1)
			Early-BC (36)	Exon11 (3), Exon18 (1)	FS (3), NS (1)
Chen et al^21^	2010	PCR-SSCP-SEQ	HBC (12)	Exon11 (2)	FS (2)
Zhang et al^23^	2011	PCR-SEQ	HBC (409)	Exon11 (12), Exon22 (1), Intron21 (1), Exon24 (2)	FS (11), NS (2), SS (2), MS (1)

Abbreviations: HBC, hereditary breast cancer; HBOC, hereditary breast/ovarian cancer; Early-BC, early-onset breast cancer; SBC, sporadic breast cancer; Bi-BOC, bilateral breast/ovarian cancer; OC, ovarian cancer; FS, frameshift mutation; NS, nonsense mutation; SS, splice-site mutation; MS, missense mutation; PCR, polymerase chain reaction; SSCP, single-strand conformation polymorphism; SEQ, sequencing; DHPLC, denaturing high-performance liquid chromatography.

**Table 2.** Disease-associated *BRCA2* germline mutations in Chinese with breast/ovarian cancer

Author (reference)	Year published	Techniques	Types of cases (No. of cases)	Mutation positions (No. of cases)	Mutation types (No. of cases)
Li et al^7^	1999	PCR-SSCP-SEQ	HBC (18)	Exon11 (3)	FS (3)
Zhi et al^9^	2002	PCR-SSCP-SEQ	HBC (16)	Exon16 (1)	FS (1)
Suter et al^11^	2004	PCR-SSCP-SEQ	HBC + SBC (645)	Exon7 (1), Exon10 (2), Exon11 (5)	FS (8)
Huang et al^14^	2008	PCR-DHPLC-SEQ	HBC (19)	Exon10 (1), Exon11 (3)	FS (3), NS (1)
Ma et al^22^	2008	PCR-DHPLC-SEQ	HBC (25)	Exon10 (1), Exon11 (2)	FS (1), NS (2)
Thirthagiri et al^15^	2008	PCR-SEQ	HBOC (78) + Early-BC (40)	Exon10 (2), Exon11 (4), Exon23 (1), Exon17 (1)	FS (6), NS (1), SS (1)
Li et al^16^	2008	PCR-DHPLC-SEQ	HBC (241)	Exon10 (2), Exon11 (5), Intron17 (1), Exon18 (1), Exon22 (2)	FS (10), SS (1)
			Early-BC (207)	Exon5 (2), Exon10 (3), Exon11 (4), Exon21 (1)	FS (9), NS (1)
Zhou et al^18^	2009	PCR-DHPLC-SEQ	HBC (17)	Exon11 (1)	FS (1)
Kwong et al^19^	2009	PCR-SEQ	High-risk BC + OC (119)	Exon10 (1), Exon11 (8), Exon15 (2), Exon18 (2), Intron16 (1)	FS (6), NS (7), SS (1)
Li et al^21^	2010	PCR-SSCP-SEQ	HBC (12)	Exon11 (1)	FS (1)
Zhang et al^23^	2011	PCR-SEQ	HBC (409)	Exon3 (1), Exon10 (4), Exon11 (13), Exon17 (1), Intron18 (1), Exon19 (2), Exon22 (1), Exon23 (3), Exon25 (1)	FS (19), NS (7), SS (1)

Abbreviations: HBC, hereditary breast cancer; HOBC, hereditary breast/ovarian cancer; Early-BC, Early-onset breast cancer; SBC, sporadic breast cancer; OC, ovarian cancer; FS, frameshift mutation; NS, nonsense mutation; SS, splice-site mutation; PCR, polymerase chain reaction; SSCP, single-strand conformation polymorphism; SEQ, sequencing; DHPLC, denaturing high-performance liquid chromatography.

The overall *BRCA1*/*BRCA2* mutation rate is relatively low in the Chinese population. In reporting mutations, most studies did not carefully distinguish familial breast cancer from early-onset breast cancer. Therefore, to analyze mutation rate we selected the 2 largest well-designed studies.^16^^,^^23^ The mutation rates of *BRCA1* and *BRCA2* in familial breast cancer ranged from 3.9% to 6.9% and 5.8% to 6.6%, respectively. This is consistent with rates in other Asian ethnic groups.^26^ Moreover, in early-onset breast cancer, mutation rates of these genes were 5.1% and 4.0%, respectively.^16^ The overall *BRCA1*/*BRCA2* mutation rate was 23.0% to 26.1% in both early-onset breast cancer and familial cases.^16^^,^^23^ A history of ovarian cancer was associated with a higher *BRAC1*/*BRCA2* mutation rate in breast cancer, as compared with patients without such a history (26.7% vs 11.9%, *P* = 0.11). Moreover, in families with 2, 3, or 4 breast or ovarian cancer cases, there was no difference in *BRAC1/BRCA2* mutations, and the mutation rates were 11.6%, 11.1%, and 11.1%, respectively. Another study, by Suter et al,^11^ found that the rates of *BRCA1* and *BRCA2* mutations in sporadic breast cancers were 0.7% (4/590) and 1.0% (6/590), respectively, in China.

*BRCA1* and *BRCA2* founder mutations have been identified in some populations. These mutations first occurred as common ancestors in a specific group of people and then spread as the population expanded, eg, the *BRCA1* 185delAG and 5382insC mutations and *BRCA2* 6174delT mutation in the Jewish population,^27^ the *BRCA2* 999del5 mutation in the Icelandic population,^28^ the *BRCA1* R71G mutation in the Spanish population,^29^^,^^30^ the *BRCA1* 4184del4 and *BRCA2* 2157delG mutations in the English population,^31^ and the *BRCA2* S2834X and 5802del4 mutations in the Japanese population.^32^ In mutation screening of these populations, screening for founder mutations should be done first. Other sites can be then screened if founder mutations are not found. This method has obvious advantages, such as shorter detection time, and lower cost, than whole-genome screening. The most common recurrent mutations in Chinese are 5589del8 in *BRCA1* and 3109C>T in *BRCA2* (Table 3). In addition, founder mutations might exist in the Chinese population, such as the 1100delAT and 5589del8 mutations in *BRCA1*^16^ and the 3109C>T mutation in *BRCA2*.^19^ Among these, the 1100delAT mutations have also been identified in Malaysian Chinese,^15^ and the 5899del8 mutation has also been reported.^11^^,^^13^ Kwong et al^19^ found that the *BRCA2* 3109C>T mutation accounted for 28.6% of all *BRCA2* mutations in a high-risk breast cancer group. Haplotype analysis confirmed that *BRCA2* 3109C>T is a founder mutation in the Southern Chinese population. However, such mutations have not been reported in other studies.

**Table 3.** Recurrent germline mutations of *BRCA1* and *BRCA2* in the Chinese population

Mutation^a^ (references)	Exon	AA change	Times reported	Frequency^b^ (%)
BRCA1				
5589del8^11^^,^^13^^,^^16^	Exon24	Stop1826	11	10.7
1100delAT^15^^,^^16^	Exon11	Stop328	6	5.8
3478del5^13^^,^^16^^,^^17^^,^^23^	Exon11	Stop1138	5	4.9
1235G>A^12^^,^^13^^,^^23^	Exon11	W372X	3	2.9
3712insG^20^	Exon11	Stop1218	3	2.9
IVS3-2A>G^11^	Intron2	Splicing site	2	1.9
IVS8-24del10^7^	Intron7	Splicing site	2	1.9
2229delAA^11^^,^^16^	Exon11	Stop710	2	1.9
3887delAG^17^^,^^23^	Exon11	Stop1265	2	1.9
4035delTT^23^	Exon11	Stop1328	2	1.9
IVS21+1delG^17^^,^^23^	Intron21	Splicing site	2	1.9
5482G>T^17^^,^^23^	Exon22	G1788V	2	1.9
5587-1del8^17^^,^^23^	Exon24	Stop1831	2	1.9
5640delA^16^^,^^23^	Exon24	Stop1842	2	1.9
BRCA2				
3109C>T^19^	Exon11	Q1037X	4	4.4
2060C>A^23^	Exon10	S611X	3	3.3
6819delTG^23^	Exon11	Stop2201	3	3.3
9326insA^15^^,^^23^	Exon23	Stop3042	3	3.3
2001del4^15^^,^^22^	Exon10	Stop612	2	2.2
2670delC^7^^,^^16^	Exon11	Stop824	2	2.2
3423del4^16^^,^^23^	Exon11	Stop1075	2	2.2
5804del4^18^^,^^23^	Exon11	Stop1862	2	2.2
5950delCT^11^^,^^16^	Exon11	Stop1091	2	2.2
6092C>G^23^	Exon11	Stop1955	2	2.2
IVS17+1G>A^15^^,^^16^	Intron17	Splicing site	2	2.2
8628del4ins5^23^	Exon19	Stop2811	2	2.2
9048del4^16^^,^^23^	Exon23	Stop2974	2	2.2

^a^GenBank reference sequences: *BRCA1* version #U14680.1; *BRCA2* version #U43746.1.

^b^Frequency of recurrent germline mutations in gene total mutations; number of total mutations: 103 in *BRCA1*, 91 in *BRCA2*.

### 2.2 Large genomic rearrangements

Conventional PCR can only detect small fragments or a single base change. The overall detection rate for PCR in detecting *BRCA1* and *BRCA2* mutations in high-risk families with breast/ovarian cancer is considerably lower than that for linkage analysis.^33^ Therefore, researchers speculate that there might be other types of mutations. Large genomic rearrangements in *BRCA1* and *BRCA2* have recently been confirmed in a number of populations. These mutations were mainly detected by multiplex ligation-dependent probe amplification (MLPA) and account for 4% to 28% of all *BRCA1*/*BRCA2* mutations.^34^ Although no relevant research in mainland China has been reported, studies of Chinese populations in other regions and countries reported large genomic rearrangements in these 2 genes. Yap et al^35^ first performed MLPA analysis using the P002 test kit to study *BRCA1* in 108 high-risk Singaporean Chinese patients and found large genomic rearrangements in 2 cases. They then used the P087 MLPA kit to reanalyze all results and found that 1 case was false-positive and that the other case had a confirmed exon 13 duplication (g.41 220_49682dup8463). Lim et al^36^ investigated 87 Singapore Chinese women at high risk for breast/ovarian cancers. Exon 13 duplication and exon 4_11a duplication (g.8730_24909dup16180) rearrangements were detected in *BRCA1* and *BRCA2*, respectively. Kwong et al^19^^,^^37^ studied the Hong Kong Chinese population and found *BRCA1* exon 17_20 4987_5277del291, exon 1_12 deletion, and *BRCA2* exon 21 8633_8754del112. Kang et al^38^ studied Chinese Malaysian families and found *BRCA1* exon 1_14 deletion in 1 case with hereditary breast/ovarian cancer, with a 78 500 bp deletion.

### 2.3 Non-coding regions and single nucleotide polymorphism

There have been reports of mutations in non-coding regions. Wang et al^39^ reported an 118A>T mutation in the 5′-untranslated region of the *BRCA1* gene in sporadic breast cancers. This mutation might downregulate translational efficiency of the protein. Chen et al^40^ detected an upstream promoter sequence of −397–+123 bp in *BRCA2* in 357 Chinese Han patients with familial or early-onset breast cancer. No pathogenic mutation was found. Chan et al^41^ reported that in a Chinese population the *BRCA1* promoter region polymorphism rs11655505 significantly reduces breast cancer risk in genotypes CT/TT (OR = 0.64, 95% CI = 0.47–0.88, *P* = 0.005) as compared with the CC genotype. However, Verderio et al^42^ did not find evidence of an association between rs11655505 and breast cancer risk in whites. In analyzing the above data, we believe that the differing results of these 2 studies may be related to the different frequency distribution of polymorphisms in Han Chinese and whites.

### 2.4 Models for predicting *BRCA1*/*BRCA2* mutations

Most reports regard *BRCA1*/*BRCA2* testing as standard for high-risk groups with early-onset or familial breast cancer. In addition, such screening is sometimes recommended for women with a history of ovarian cancer. However, the sensitivity and specificity of these standards are a concern. For example, in China, *BRCA1*/*BRCA2* testing is unnecessary in more than 80% of so-called high-risk groups. Currently, there are some international models for predicting *BRCA1*/*BRCA2* gene mutations, such as the BRCApro, BOADICEA, and Myraid models, of which BRCApro is the most commonly used model for familial breast cancer.^43^ The BOADICEA model can be used for both familial breast cancer and age-specific breast cancer in small families.^44^ The Myriad model, developed by Myriad Genetics, Inc. in the United States, is based on data from 10 000 cases of breast/ovarian cancer, including 2539 Jews.^45^ In the Italian population, the Myriad model has better sensitivity than the BRCApro model for predicting *BRCA1*/*BRCA2* mutations (89% vs 67%, respectively), and similar specificity (51% vs 57%, respectively).^46^ Thirthagiri et al^15^ studied 187 high-risk breast cancer patients in Asia (63.1% of whom were Chinese) and showed that the sensitivity of the BOADICEA prediction model for *BRCA1* and *BRCA2* mutations was 57% and 9%, respectively. Rao et al^47^ applied the BRCApro, Penn, and Myriad models to 212 Chinese familial breast cancer patients, among whom 33 had *BRCA1* or *BRCA2* mutations. The sensitivity of the 3 models for predicting *BRCA1* mutation was 67%, 13%, and 40%, respectively, while the sensitivity of the BRCApro and Penn models for predicting *BRCA2* mutation was 26% and 1%, respectively. Clearly, these models are not suitable for the Chinese population, probably because the penetrance and prevalence parameters of these models were derived from whites.^48^ Chen et al incorporated Asian-specific phenocopy rates into the BRCApro model. The modified model was able to predict more mutations, especially in the lowest decile, as compared with the unmodified BRCApro model.^49^ Therefore, Rao et al^50^ established a mutation prediction model based on the characteristics of *BRCA1*- or *BRCA2*-associated breast cancers in the Chinese population. It included 4 significant variables: age at onset of the youngest breast cancer cases in a family, age at onset of breast cancer in the index patient, presence of stomach cancer in a family, and presence of ovarian cancer in a family. The model had a relatively high predictive power, as compared with the BRCApro, Couch, and Sh-E models, in the Chinese population.

### 2.5 Clinicopathologic characteristics of *BRCA*-associated breast cancer

The clinicopathologic features of *BRCA*-associated breast cancer are more specific than those of sporadic breast cancer. In the Chinese population, as compared with sporadic breast cancer, *BRCA1*-associated breast cancer had a higher pathologic grade, and higher percentages of CerbB-2-negative and triple negative (ER/PR/CerbB-2-negative) tumors. However, there was no significant difference in clinical stage, tumor size, lymphatic metastasis, rate of medullary carcinoma development, or estrogen receptor (ER) or progesterone receptor (PR) positivity.^17^^,^^51^ Kwong et al^52^ reported that *BRCA* mutation carriers were more likely than non-carriers to have cancers of higher histologic grade and triple negative cancers (48.3% vs 25.6%, *P* = 0.01). As compared with *BRCA2*-associated breast cancer, *BRCA1*-associated breast cancer had a higher ER-negative rate and larger tumor diameter. Li et al^16^ reported that age at onset of *BRCA1*-associated breast cancer was significantly earlier as compared with *BRCA2*-associated and sporadic breast cancers (median age: 36.1 years in *BRCA1*-associated, 42.7 years in *BRCA2*-associated, and 47.3 years in sporadic breast cancer). However, there was no significant difference in age at onset in patients with *BRCA2*-associated and sporadic breast cancers. *BRCA*-associated breast cancer pedigrees showed that gastric cancer was the most common cancer, other than breast/ovarian cancer. In some populations, *TP53* mutations have been detected in 56% to 100% of *BRCA1*-associated breast cancer cases,^53^ but detection of *TP53* mutations in the Chinese population has not been reported.

## 3. OTHER BREAST CANCER SUSCEPTIBILITY GENES

In addition to *BRCA1* and *BRCA2*, other breast cancer susceptibility genes have also been extensively studied in Chinese. In Table 4, we summarize all studies of disease-associated germline mutations in Chinese women with *BRCA1*/*BRCA2*-negative breast cancer.

**Table 4.** Germline mutations of other genes in Chinese with *BRCA1*/*BRCA*2-negative breast cancer

Gene	Location	Disease	Author	Techniques	Cases (No.)	Analysis	Mutation (No.)	Clinical value
*TP35*	17p13.1	Li-Fraumeni syndrome	Cao et al^58^	DHPLC	EBC + FBC (240)	Whole gene	563T>C (1)	Yes
							643del18 (1)	Yes
*BRIP1*	17q22.2	Fanconi anemia	Cao et al^61^	DHPLC	EBC + FBC (357)	Whole gene	Q944E (2)	Uncertain
*PALB2*	16p12.1	Fanconi anemia	Cao et al^64^	DHPLC	EBC + FBC (360)	Whole gene	751C>T (2)	Yes
							1050del2insTCT (1)	Yes
*CDH1*	16q22.1	Hereditary diffuse gastric cancer/lobular breast cancer	Zhu et al^69^	SSCP	FLBC (1)	Whole gene	A154A (1)	Uncertain
*CHEK2*	22q12.1	Li-Fraumeni syndrome	Chen et al^72^	Direct SEQ	FBC (74)	1100delC	None	No
			Song et al^73^	Direct SEQ	EBC + FBC (117)	1100delC	None	No
			Thirthagiri et al^74^	RFLP	EBC + FBC + SBC (452)	1100delC	None	No
			Liu et al^75^	DHPLC	FBC (118)	Whole gene	H371Y (5)	Yes
*RAD50*	5q23–31	Louis-Bar syndrome	He et al^89^	DHPLC	EBC + FBC (384)	Whole gene	None	No
*NBS1*	8q21–24	Nijmegen breakage syndrome	He et al^89^	DHPLC	EBC + FBC (384)	Whole gene	None	No

Abbreviations: DHPLC, denaturing high-performance liquid chromatography; SSCP, single-strand conformation polymorphism; SEQ, sequencing; RFLP, restriction fragment length polymorphism; EBC, early-onset breast cancer; FBC, familial breast cancer; FLBC, familial lobular breast cancer; SBC, sporadic breast cancer; No., number.

### 3.1 *TP53*

The protein of the *TP53* gene has important roles in the control of cell cycle progression, repair of DNA damage, genomic stability, and apoptosis.^54^ *TP53* mutations are found in 50% to 70% of individuals with Li-Fraumeni syndrome, an autosomal dominant inherited disease that was first reported in 1969 and is a rare cancer-predisposing syndrome. The most common cancers associated with this syndrome include breast cancer, leukemia, soft tissue sarcoma, and brain tumors. Li-Fraumeni syndrome accounts for about 1% of hereditary breast cancers.^55^ Age at tumor onset is earlier: the risk of developing cancer by age 40 years is 50% and as high as 90% by age 60.^56^ In 24 families with Li-Fraumeni syndrome, the incidence of breast cancer was 22.5% (45/200), age at onset was younger than 45 years in 77% of women, the proportion of bilateral breast cancer was 5%, and 11% of breast cancer cases were associated with other tumors.^57^ In China, Cao et al^58^ found that the frequency of disease-associated *TP53* germline mutations was 0.8% (1/240). *BRCA1*/*BRCA2* mutations were excluded in these patients, and the clinical criteria were not consistent with Li-Fraumeni/Li-Fraumeni-like syndrome. It had been proposed that *TP53* should be tested in women without *BRCA1*/*BRCA2* mutation who are at high risk for breast cancer.

### 3.2 *BRIP1*

The *BRIP1* gene is also known as *BACH1*. Biallelic mutation carriers of this gene are susceptible to Fanconi anemia. The BACH1 protein binds with the BRCT protein binding sites and has a key role in repair of DNA double-stranded breaks via the BRCA1 pathway.^59^ A study of a large sample of 1212 patients found truncating mutations in 9 breast cancer patients from *BRCA1*/*BRCA2* mutation-negative families, while such mutations were found in only 2/2081 healthy individuals, suggesting that *BRIP1* heterozygous mutation carriers had a relative risk of 2.0 for breast cancer.^60^ Cao et al^61^ suggested that the Q944E alteration represents a rare disease-related allele in Chinese non-*BRCA1*/*BRCA2* early-onset or familial breast cancer. However, due to lack of functional verification it is unclear whether this mutation causes protein dysfunction. In addition, Huo et al^62^ used PCR-primer introduced restriction analysis assays to genotype the rs4986764 polymorphism of BRIP1 in a case-control study of 568 breast cancer cases and 624 controls in a Chinese population. They confirmed that the polymorphism had no relationship with breast cancer risk. In sum, germline mutations of *BRIP1* are extremely rare in Chinese, and there is thus insufficient evidence for recommending *BRIP1* in genetic testing of Chinese.

### 3.3 *PALB2*

Like the *BRIP1* gene, individuals with biallelic mutations in *PALB2* are susceptible to Fanconi anemia. The *PALB2* protein can bind to the N-terminal of the BRCA2 protein and has an important role in DNA stability. Rahman et al^63^ reported truncating *PALB2* mutations in 10/923 individuals with familial breast cancer and no such mutations in healthy controls, suggesting that such mutations conferred a relative risk of 2.3 for breast cancer. Nevertheless, the phenomenon of incomplete segregation of the mutation reflected difficulties in screening this gene in breast cancer families when unaffected women asked for predictive molecular testing to determine their individual breast cancer risk. Cao et al^64^ found that the frequency of disease-related germline mutations in *PALB2* was 0.8% (3/360) in *BRCA1*/*BRCA2*-negative Chinese women with early-onset or familial breast cancer. Chen et al^65^ used the GenomeLab SNPstream 12-plex Genotyping System to study SNPs of *PALB2* in a cohort of 1049 breast cancer cases and 1073 controls in a Chinese population. They confirmed that rs249954, rs120963, and rs16940342 were associated with 36% (*P* = 0.001; TT/TC vs CC genotypes), 25% (*P* = 0.014; CC/CT vs TT genotypes), and 21% (*P* = 0.037; GG/GA vs AA genotypes) increases in breast cancer risk, respectively, while rs249935 was not associated with breast cancer risk. However, Cao et al^66^ reached a different conclusion. They found that the *PALB2* rs249935 G allele was related to a 1.21-fold increased risk of breast cancer for each A allele carried, while rs249954 and rs16940342 had no role in breast cancer risk. In addition, they found that, as compared with rs447529 CC homozygotes, carriers of the GG/GC genotypes had a 0.43-fold lower risk of breast cancer. The completely different results of these 2 studies could be due to case selection bias and thus need to be validated in other populations. Therefore, germline mutations in *PALB2* should be tested in Chinese at high risk for breast cancer. However, it is unclear whether SNPs are within the scope of genetic counseling.

### 3.4 *CDH1*

The *CDH1* gene encodes E-cadherin, the calcium-dependent cell–cell adhesion glycoprotein. *CDH1* gene mutation is related to hereditary diffuse gastric cancer and lobular carcinoma of the breast. The risk of breast cancer was 50% higher in women with a family history of diffuse gastric breast cancer.^67^ *CDH1* mutations have also been found to be associated with familial lobular carcinoma of the breast in individuals without a family history of diffuse gastric breast cancer.^68^ The *CDH1* gene appears to be rare in Chinese with breast cancer. Zhu et al^69^ found no germline disease-associated mutations in a patient with familial diffuse gastric cancer and lobular breast cancer. More research is required in order to describe the role of *CDH1* mutations in Chinese at high risk of breast cancer.

### 3.5 *CHEK2*

The *CHEK2* gene encodes a cell cycle checkpoint kinase. When DNA is damaged, CHEK2 is activated by ATM, resulting in phosphorylation of BRCA1, which has a role in the repair of DNA double-stranded breaks. The *CHEK2* 1100delC mutation has been found to double the risk of breast cancer in women.^70^ A meta-analysis found a cumulative risk of 37% before age 70 years in carriers of the mutation.^71^ However, the *CHEK2* 1100delC mutation was not found in Chinese women at high risk of breast cancer,^72^^–^^75^ or in other Asian populations.^76^ Liu et al^75^ screened the entire gene of CHEK2 and found that H371Y increased breast cancer risk (OR = 2.43, 95% CI = 1.07–5.52, *P* = 0.034). On the basis of these studies, we believe that the *CHEK2* 1100delC mutation is either rare or absent in high-risk breast cancer groups in China. The incidence of this mutation in breast cancer is very low; therefore, *CHEK2* 1100delC is not an appropriate indicator in breast cancer gene mutation screening. However, the H371Y mutation confers a moderate risk of breast cancer in Chinese women and should be considered in genetic testing.

### 3.6 *PTEN*

The *PTEN* gene codes a dual-specificity phosphatase with lipid and protein phosphatase activity.^77^ Mutations in the *PTEN* gene cause Cowden syndrome, a rare autosomal dominant inherited disease that predisposes affected individuals to breast cancer, thyroid carcinoma, endometrial carcinoma, and hamartoma with high fat content.^78^ The risk of developing breast cancer before age 70 years is 30% to 50% in affected women, and most cases occur before menopause. There is also an increased risk of breast cancer in men.^79^ When Cowden syndrome was diagnosed according to Consortium criteria for the diagnosis of Cowden syndrome, nearly 80% of patients carried *PTEN* mutations.^80^ Yang et al^81^ reported *PTEN* mutations in 22% (11/50) of breast cancers in China, but the germline mutations have not been studied.

### 3.7 *ATM*

The protein expressed by the *ATM* gene plays a role in DNA double-stranded break repair pathways by upstreaming the *BRCA1* gene. Biallelic mutations of the *ATM* gene cause ataxia telangiectasia, which manifests as cerebellar ataxia, immune deficiency, and a variety of tumors such as leukemia, lymphoma, glioma, medulloblastoma, and breast cancer. The role of *ATM* as a risk factor for breast cancer has been discussed for nearly 20 years, and the estimated relative risk is 1.3 to 12.7.^82^ Renwick et al^83^ reported *ATM* mutations in 12 of 441 breast cancer cases with *BRCA1*/*BRCA2*-negative mutations. The mutation rate was 0.38% (2/521) in healthy controls, suggesting a relative risk of 2.37 for breast cancer. Currently, no germline mutations have been found in the Chinese population, and only a small number of *ATM* SNPs have been reported as risk factors for breast cancer. In a 2-stage case-control study Ye et al^84^ reported that the *ATM* polymorphisms rs1800054, rs1800058, rs664143, rs228589, and rs1003623 had no role in breast cancer. Wang et al^85^ studied 360 samples from Han Chinese and reported that, by interacting with *BRCA1* rs4793191 and *BRCA2* rs9567623, the *ATM* polymorphism rs611646 might have a role in breast cancer risk. It remains to be confirmed in a germline mutations study whether *ATM* is the breast cancer susceptibility gene in Chinese. The role of the gene SNP in increasing breast cancer risk has not been widely studied. There is no basis for clinical *ATM* genetic counseling at this time.

### 3.8 *RAD50* and *NBS1*

The proteins of the genes *NBS1*, *RAD50*, and *MRE11* form MRN complex, which has a role in the identification and repair of DNA double-stranded breaks. Mutations in the *NBS1* gene cause Nijmegen breakage syndrome, an autosomal recessive inherited disease that manifests as microcephaly, growth retardation, immunodeficiency, and cancer susceptibility. In some ethnic groups *NBS1* and *RAD50* have been shown to be associated with breast cancer, eg, *NBS1* 657del5 increased breast cancer risk by 3-fold in Central and Eastern Europeans,^86^ I171V increased breast cancer risk among Poles,^87^ and *RAD50* 687delT was associated with a relative risk of 4.3 for breast cancer.^88^ However, no deleterious mutation in these 2 genes was found in 384 non-*BRCA1/BRCA2* hereditary breast cancers in Chinese.^89^ Thus, there is no evidence to recommend *RAD50* and *NBS1* for genetic testing in China.

## 4. CONCLUSIONS

*BRCA1* and *BRCA2* are the most important genetic factors in hereditary breast cancer in the Han Chinese population, but the mutation rates are lower than in other ethnic groups. Future efforts should emphasize development of a model that predicts *BRCA1*/*BRCA2* mutation, so as to avoid unnecessary mutation-negative genetic testing. The characteristics of *BRCA1*/*BRCA2* mutations in the Chinese population suggest that screening may detect founder mutations. Subsequent founder mutation-negative cases could be detected in exon 11. If all the above results are negative, other exons should be tested. This process can improve detection efficiency and reduce the overall cost of testing. More research on non-coding regions is needed. Also, as in other populations, large genomic rearrangements warrant the attention of researchers in mainland China.

Although germline mutations in *TP53* and *PALB2* are rare, Chinese with *BRCA1*/*BRCA2* mutation-negative high-risk breast cancer should be screened for these 2 genes. Moreover, *CHEK2* H371Y should be considered in genetic testing. *CHEK2* 1100delC, *RAD50*, and *NBS1* do not appear to correlate with breast cancer in the Chinese population and should not be considered in genetic testing. There is a need for comprehensive studies of germline mutations in other genes, such as *CDH1*, *PTEN*, *ATM*, and *BRIP1*. In addition, 2 other high penetrance breast cancer susceptibility genes—*STK11* and *NF1*—have not been reported in the Chinese population. These genes cause autosomal dominant hereditary diseases such as Peutz–Jeghers syndrome and neurofibromatosis I, respectively, and need to be carefully investigated. The role of SNPs of these genes in breast cancer is not clear. However, at present, these genes have no value in genetic counseling for breast cancer risk in China. In total, susceptibility genes explain breast cancer etiology in less than 20% of Chinese women at high risk of breast cancer. Further studies are needed so as to identify other susceptibility genes. The development of new technologies, such as next generation sequencing and proteomics technology, has led to new ideas in the search for new susceptibility genes.

The main purpose of research on hereditary breast cancer is to identify high-risk individuals and develop early interventions, including intensive clinical examination and preventive measures that reduce breast cancer morbidity and mortality. However, preventive intervention reports on individuals at high risk of breast cancer are limited in the Chinese population. Thus, in China, and especially in mainland Chinese patients and clinicians, there is insufficient understanding of the importance of genetic screening and counseling. Therefore, comprehensive genetic counseling agencies for patients should be improved, to give them the necessary clinical, social, and psychological support. Research on hereditary breast cancer in China still has a long road ahead.

## ONLINE ONLY MATERIALS

eTables 1. Disease-associated *BRCA1* germline mutations in Chinese women with high-risk breast cancer.

eTables 2. Disease-associated *BRCA2* germline mutations in Chinese women with high-risk breast cancer.
