# Supplementary material for: Hereditary Breast Cancer in the Han Chinese Population
Source: J Epidemiol. 2013 Mar 5;23(2):75–84. doi: 10.2188/jea.JE20120043 (PMC3700245; doi:10.2188/jea.JE20120043)
Supplement: eTables 1. — Disease-associated BRCA1 germline mutations in Chinese women with high-risk breast cancer. [file je-23-075-s001.pdf]

eTable 1. Disease-associated *BRCA1* germline mutations in Chinese women with high-risk breast cancer

Mutation[a] [references]	Exon	AA change	Times reported	BIC
5589del8[11, 13, 16]	Exon24	Stop1826	11	BIC
1100delAT[15, 16]	Exon11	Stop328	6	BIC
3478del5[13, 16, 17, 23]	Exon11	Stop1138	5	None
1235G＞A[12, 13, 23]	Exon11	W372X	3	BIC
3712insG[20]	Exon11	Stop1218	3	None
IVS3-2A＞G[11]	Intron2	Splicing site	2	None
IVS8-24del10[7]	Intron7	Splicing site	2	None
2229delAA[11, 16]	Exon11	Stop710	2	BIC
3887delAG[17, 23]	Exon11	Stop1265	2	None
4035delTT[23]	Exon11	Stop1328	2	BIC
IVS21+1delG[17, 23]	Intron21	Splicing site	2	None
5482G＞T[17, 23]	Exon22	G1788V	2	BIC
5587-1del8[17, 23]	Exon24	Stop1831	2	None
5640delA[16, 23]	Exon24	Stop1842	2	BIC
185insA[15]	Exon2	Stop40	1	BIC
IVS3+1C＞T[15]	Intron3	Splicing site	1	None
IVS3+2delT[15]	Intron3	Splicing site	1	None
IVS3+2T＞G[16]	Intron3	Splicing site	1	BIC
IVS5-12A＞G[15]	Intron4	Splicing site	1	None

307T＞A[20]	Exon5	L63X	1	BIC
331G＞A[18]	Exon5	R71K	1	BIC
IVS5+1G＞T[19]	Intron5	Splicing site	1	BIC
339C＞T[14]	Exon6	Q74X	1	BIC
470delCT[19]	Eoxn8	S157X	1	BIC
582C＞T[16]	Exon8	Q115X	1	None
589delCT[15]	Exon8	Stop157	1	BIC
667delG[13]	Exon9	Stop184	1	None
735C＞T[16]	Exon10	Q206X	1	None
1010delA[19]	Exon11	Stop340	1	BIC
1129insA[23]	Exon11	Stop345	1	BIC
1398G＞T[16]	Exon11	E427X	1	BIC
1510delC[8]	Exon11	Stop474	1	None
1523delG[8]	Exon11	Stop475	1	None
1584C＞T[9]	Exon11	E489X	1	None
1779G＞T[23]	Exon11	E554X	1	None
1958insA[20]	Exon11	Stop625	1	None
2064G＞T[13]	Exon11	Stop649	1	BIC
2071insA[23]	Exon11	Stop672	1	BIC
2129insTG[17]	Exon11	Stop701	1	None
2171delA[20]	Exon11	Stop702	1	None
2367del5[23]	Exon11	Stop759	1	None

2418delA[23]	Exon11	Stop791	1	BIC
2430insC[8]	Exon11	Stop776	1	None
2635G＞T[19]	Exon11	E879X	1	BIC
2691C＞T[16]	Exon11	Q858X	1	None
2722C＞A[15]	Exon11	S868X	1	None
2732insT[8]	Exon11	Stop902	1	None
2790delT[16]	Exon11	Stop891	1	None
2809insA[23]	Exon11	Stop902	1	BIC
2917delGT[16]	Exon11	Stop998	1	None
3241C＞G[16]	Exon11	S1041X	1	None
3342del4[19]	Exon11	E1115X	1	None
3347A＞T[13]	Exon11	R1076X	1	None
3378delG[9]	Exon11	Stop1108	1	None
3389delAC[11]	Exon11	Stop1091	1	None
3443insA[11]	Exon11	Stop1114	1	None
3449insA[16]	Exon11	Stop1114	1	BIC
3569delT[23]	Exon11	Stop1154	1	BIC
3870del4[10]	Exon11	Stop1263	1	None
3889delAG[15]	Exon11	Stop1265	1	BIC
4046insG[19]	Exon11	Stop1355	1	None
4193insA[21]	Exon11	Stop1254	1	None
4196delA[21]	Exon11	Stop1365	1	None

4255delCT[13]	Exon12	Stop1389	1	None
4377C＞T[15]	Exon13	Q1420X	1	BIC
4446C＞T[8]	Exon13	R1443X	1	BIC
4372C＞T[19]	Exon14	Q1458X	1	BIC
5028delC[9]	Exon16	Stop1639	1	BIC
IVS17-1G＞T[16]	Intron16	Splicing site	1	None
5199G＞T[20]	Exon18	E1694X	1	BIC
IVS21+1G＞C[16]	Intron21	Splicing site	1	None

Abbreviation: BIC, Breast Cancer Information Core database.
[a] GenBank reference sequences: *BRCA1* version #U14680.1
