# Supplementary material for: Hereditary Breast Cancer in the Han Chinese Population
Source: J Epidemiol. 2013 Mar 5;23(2):75–84. doi: 10.2188/jea.JE20120043 (PMC3700245; doi:10.2188/jea.JE20120043)
Supplement: eTables 2. — Disease-associated BRCA2 germline mutations in Chinese women with high-risk breast cancer. [file je-23-075-s002.pdf]

eTable 2. Disease-associated *BRCA2* germline mutations in Chinese women with high-risk breast cancer

Mutation[a] [references]	Exon	AA change	Times reported	BIC
3109C＞T[19]	Exon11	Q1037X	4	BIC
2060C＞A[23]	Exon10	S611X	3	BIC
6819delTG[23]	Exon11	Stop2201	3	BIC
9326insA[15, 23]	Exon23	Stop3042	3	BIC
2001del4[15, 22]	Exon10	Stop612	2	BIC
2670delC[7, 16]	Exon11	Stop824	2	None
3423del4[16, 23]	Exon11	Stop1075	2	BIC
5804del4[18, 23]	Exon11	Stop1862	2	BIC
5950delCT[11, 16]	Exon11	Stop1091	2	BIC
6092C＞G[23]	Exon11	Stop1955	2	BIC
IVS17+1G＞A[15, 16]	Intron17	Splicing site	2	BIC
8628del4ins5[23]	Exon19	Stop2811	2	None
9048del4[16, 23]	Exon23	Stop2974	2	None
490delCT[23]	Exon3	Stop99	1	BIC
692del5[16]	Exon5	Stop180	1	None
698del5[16]	Exon5	Stop180	1	BIC
808delT[11]	Exon7	Stop198	1	None
1184insA[15]	Exon10	Stop326	1	BIC
1216insAA[23]	Exon10	Stop349	1	None

1261C＞T[19]	Exon10	Q421X	1	BIC
1308delA[16]	Exon10	Stop366	1	None
1527insA[16]	Exon10	Stop451	1	None
1529del4[11]	Exon10	Stop458	1	BIC
1796del5[14]	Exon10	Stop559	1	None
2000del4[11]	Exon10	Stop612	1	BIC
2041delA[16]	Exon10	Stop615	1	BIC
2109delA[16]	Exon10	Stop643	1	None
2129delC[16]	Exon10	Stop643	1	None
2372C＞G[14]	Exon11	S791X	1	None
2808del4[14]	Exon11	Stop958	1	None
2816insA[16]	Exon11	Stop880	1	BIC
2864delCT[15]	Exon11	Stop879	1	BIC
3034del4[16]	Exon11	Stop959	1	BIC
3036del4[23]	Exon11	Stop959	1	BIC
3073delT[7]	Exon11	Stop958	1	None
3391del4[11]	Exon11	Stop1058	1	None
3395del4[23]	Exon11	Stop1058	1	BIC
3972del4[16]	Exon11	Stop1257	1	BIC
4099C＞T[22]	Exon11	Q1291X	1	BIC
4563delGT[19]	Exon11	Stop1527	1	None
5164delAT[19]	Exon11	Stop1725	1	BIC

5239insT[21]	Exon11	Stop1676	1	None
5301insA[11]	Exon11	Stop1692	1	BIC
5392delAG[23]	Exon11	Stop1725	1	BIC
5399delT[23]	Exon11	Stop1740	1	None
5722delCT[19]	Exon11	Stop1909	1	BIC
5802del4[16]	Exon11	Stop1861	1	None
5851del4[19]	Exon11	Stop1961	1	BIC
5873C＞A[22]	Exon11	S1882X	1	BIC
5909insA[16]	Exon11	Stop1894	1	BIC
6261delTT[16]	Exon11	Stop2016	1	None
6275delTT[14]	Exon11	Stop2098	1	None
6553delGT[16]	Exon11	Stop2109	1	BIC
6561del5[11]	Exon11	Stop2126	1	None
6633del5[11]	Exon11	Stop2137	1	BIC
6677insTA[23]	Exon11	Stop2168	1	BIC
6696delTC[7]	Exon11	Stop2173	1	BIC
6901delA[15]	Exon11	Stop2228	1	None
6943C＞T[15]	Exon11	E2239X	1	None
7028C＞A[23]	Exon11	S2267X	1	None
7467insT[19]	Exon15	Stop2496	1	None
7471C＞T[19]	Exon15	Q2491X	1	BIC
IVS17-9T＞G[19]	Intron16	Splicing site	1	None

7883del4[9]	Exon16	Stop2646	1	BIC
8047dupGCAAAAC[19]	Exon18	Stop2695	1	None
8066delGT[19]	Exon18	Stop2691	1	BIC
8191delC[23]	Exon17	Stop2656	1	None
8462insT[16]	Exon18	Stop2763	1	None
IVS18+1G＞A[23]	Intron18	Splicing site	1	None
8827A＞T[16]	Exon21	K2882X	1	None
9178delT[16]	Exon22	Stop2987	1	None
9235G＞T[23]	Exon23	G3003X	1	None
9584delTAinsG[23]	Exon25	Stop3119	1	None

Abbreviation: BIC, Breast Cancer Information Core database.
[a] GenBank reference sequences: BRCA2 version #U43746.1
